# Development and validation of a machine learning-based risk prediction model for sarcopenia in community hospital patients: a retrospective cohort study

**DOI:** 10.3389/fragi.2026.1772792

**Published:** 2026-02-26

**Authors:** Xue Zhao, Wang Yao, Jiawei Shen, Xinyu Tang, Jue Zheng, Chang Guo, Sun Ye, Miqiong Li, Chao Wang, Peihao Yin

**Affiliations:** 1 Department of General Practice, Changshou Community Health Service Center of Putuo District, Shanghai, China; 2 Department of Oncology, Shanghai Jiaotong University School of Medicine Affiliated Ruijin Hospital, Shanghai, China; 3 Department of General Surgery, Putuo People’s Hospital, School of Medicine, Tongji University, Shanghai, China; 4 Department of Clinical Nutrition, Putuo People’s Hospital, School of Medicine, Tongji University, Shanghai, China; 5 Department of Hospital Infection Control and Prevention, Putuo People’s Hospital, School of Medicine, Tongji University, Shanghai, China

**Keywords:** machine learning, nomogram, risk prediction, sarcopenia, Shap

## Abstract

**Introduction:**

Sarcopenia, a progressive age-related loss of skeletal muscle mass and strength, represents a growing public health challenge amid global population aging. Early detection remains difficult with conventional diagnostic approaches.

**Methods:**

This study aimed to develop and validate reliable machine learning (ML) models to identify key risk factors for sarcopenia in community hospital settings. Using retrospective data from 1,650 patients at a community health center, we collected comprehensive demographic, clinical, and lifestyle variables. Twelve ML models—including Random Forest, Support Vector Machine, XGBoost, and Logistic Regression—were constructed and evaluated using 5-fold cross validation.

**Results:**

The CatBoost, LightGBM, and Gradient Boosting Decision Tree models demonstrated superior predictive performance, with area under the receiver operating characteristic curve (AUROC) values of 0.999, 0.996, and 0.995, respectively. SHapley Additive exPlanations (SHAP) analysis revealed that SARC_Cal_score, body mass index (BMI), and age belong to the most influential predictors, while a greater chronic disease burden was positively associated with sarcopenia risk.

**Conclusion:**

In conclusion, ML models show substantial potential for clinical application in identifying sarcopenia risk, thereby supporting early intervention strategies. This approach enhances detection capabilities and provides a practical tool for individualized treatment planning in community-based elderly care. Future research should integrate additional biomarkers and environmental factors to further improve model accuracy and facilitate integration into clinical workflows.

## Introduction

With the increasing global aging of the population, sarcopenia has emerged as a major public health concern affecting older adults (Kirk et al., 2024; Petermann-Rocha et al., 2022). It is characterized not only by a decline in muscle mass and strength but is also associated with various adverse health outcomes, including functional impairment, diminished quality of life, and premature death (Sayer et al., 2024). Sarcopenia exerts particularly significant effects in cancer patients. Studies indicate that sarcopenia correlates with poorer cancer prognosis and adversely affects overall survival and recurrence rates (Yoon et al., 2020). For example, in patients receiving CAR T-cell therapy, sarcopenia correlates with inferior survival outcomes (Jhaveri et al., 2025), our previous study have found that sarcopenia is associated with inflammation and immune responses in advanced CRC patients treated with fruquintinib and identified SYK gene as a risk factor for fruquintinib-caused sarcopenia via Mendelian randomization analysis (Wang et al., 2025), suggesting that maintaining muscle mass is crucial for therapeutic efficacy.

Research indicates that the onset of sarcopenia is influenced by multiple factors, such as age, malnutrition, chronic diseases, and lifestyle (Cruz-Jentoft and Sayer, 2019; Puš et al., 2025; Du et al., 2025a). Consequently, identifying risk factors associated with sarcopenia is essential for early intervention and for improving the health of the elderly population.

This study aims to identify factors associated with sarcopenia among patients in community hospitals using machine learning models. We will use retrospective cohort data from the Changshou Community Health Center to develop multiple machine learning models for predicting the onset of sarcopenia. By analyzing the clinical and demographic characteristics of 1,650 patients, we will examine variables that may affect muscle mass and strength, including age, sex, body weight, height, BMI, lifestyle, and chronic diseases (Ikeji et al., 2023). These variables were selected based on domain expertise and their potential discriminatory power in clinical practice.

The ultimate objective of this research is to establish an effective predictive model for sarcopenia and to develop a corresponding risk assessment tool for clinical use. This tool will provide a scientific basis for the early identification and management of sarcopenia, thereby potentially improving the quality of life and health outcomes of older patients. Through an in-depth analysis of sarcopenia-related factors, this study aims to provide new insights and methods for future research and clinical practice, promoting early detection and effective management of the condition.

## Methods

### Study population

This retrospective cohort study developed machine learning models to predict sarcopenia in community hospital patients. This study was approved by Institutional Review Board of Changshou Community Health Center and the Ethics Committee of Putuo People’s Hospital of Shanghai (Number:2025006), registered at the Chinese Clinical Trial Registry (Registration No.: ChiCTR2500099224, https://www.chictr.org.cn/) and filed in the Chinese Medical Research Registration and Filing System (MR-31-25-022873). Meanwhile, the patients’ informed consent was acquired. This study adhered to the TRIPOD reporting standards for transparent reporting of multivariable prediction models in individual prognosis or diagnosis.

Sarcopenia was diagnosed based on the 2019 consensus criteria of the Asian Working Group for Sarcopenia (AWGS) (Chen et al., 2020). The diagnosis required the co-presence of low muscle mass and low muscle strength. Low muscle mass was defined as a skeletal muscle mass index (SMI) below the sex-specific AWGS 2019 cut-offs: <7.0 kg/m^2^ for men and <5.7 kg/m^2^ for women. Low muscle strength was defined as handgrip strength <28 kg for men and <18 kg for women.

The dataset was obtained from the Changshou Community Health Center, Six patients with incomplete or missing records were excluded, 1650 patients were included in this study. The Synthetic Minority Oversampling Technique (SMOTE) was employed to address class imbalance (Du et al., 2024), and patients were randomly allocated into training and test sets at a 7:3 ratio using computer-generated randomization.

### Predictor variables

The variables selected to predict the occurrence of sarcopenia included various demographic and clinical variables. These 35 variables were based on domain expertise, and features with potential discriminative power to predict sarcopenia in clinical practice. Demographic variables included gender, age, weight, height, BMI, marriage status, educational experience, living condition, pension, sleep duration, outdoor activity time, living floor, living area, smoking, drinking, building with elevator or not, coffee and tea, physical exercise and teeth. Clinical variables included hypertension, diabetes, COPD, hyperuricemia, osteoarthritis, osteoporosis, tumor, CKD, other disease, chronic disease number, drug amount, MNA-SF score, GLIM phenotypic criteria, GLIM etiologic criteria, GLIM-malnutrition, and SARC-Calf score.

### Machine learning models and evaluation

Twelve machine learning models: random forest learner (RF), k-nearest neighbour learner (K-NN), support vector machine learner (SVM), extreme gradient boosting learner (XGBoost), light gradient boosting machine learner (LightGBM), CatBoost learner, naïve bayes learner, Decision tree learner, gradient boosting decision tree learner, Adaboost, logistic classification learner, multilayer perceptron learner were constructed, and in model development and comparison (Du et al., 2025b), we utilized 5-fold cross-validation, which provides a robust and reliable method for evaluating model performance metrics.

Each model was evaluated using multiple performance metrics: precision, recall, accuracy, F1 score, prevalence, specificity, false negative rate (FNR), false positive rate (FPR), matthews correlation coefficient (MCC), and the area under the receiver operating characteristic curve (AUROC). Accuracy represented the proportion of correctly classified instances among all observations. Precision denoted the ratio of true positive predictions to all positive predictions, while recall indicated the ratio of true positives to all actual positive instances. The F1 score was the harmonic mean of precision and recall. Prevalence referred to the proportion of actual positive instances in the dataset. Specificity measured the ratio of true negatives to all actual negative instances. FNR represented the proportion of actual positives incorrectly predicted as negatives, while FPR indicated the proportion of actual negatives incorrectly predicted as positives. MCC provided a balanced measure of classification quality, calculated as a correlation coefficient between observed and predicted binary classifications. Finally, the AUROC curve graphically represents the trade-off between the true positive rate (recall) and the false positive rate (1 - specificity) across different classification thresholds.

### SHAP

“Shapviz” is an R package designed to interpret machine learning model predictions by providing visual explanations based on SHAP (SHapley Additive exPlanations) values. SHAP values quantify the contribution of each feature to individual predictions, indicating either positive or negative effects. The package includes a feature importance plot, which ranks features by the average magnitude of their SHAP values, thus highlighting those with the greatest influence on the model’s output. In this study, SHAP was used to identify which variables play an important role in the prediction of sarcopenia.

The top five contributing variables selected by SHAP in CatBoost, LightGBM and GBDT models were involved in logistic regression model and established a nomogram to predict sarcopenia. Calibration was employed to evaluate the performance of the aforementioned nomogram models. A calibration plot, which visually represents the relationship between predicted and actual risk, was constructed using bootstrap resampling methods. On these plots, a 45-degree diagonal line indicates excellent absolute risk estimation. Net Reclassification Improvement (NRI) and Integrated Discrimination Improvement (IDI) were both employed to compare the discriminative ability between the models based on CatBoost, LightGBM and GBDT contributing variables.

### Statistical analysis

Continuous variables with a normal distribution were presented as mean ± standard deviation and compared using the independent samples *t*-test, whereas non-normally distributed variables were expressed as median with interquartile range (IQR) and analyzed with the Mann-Whitney *U* test. Categorical variables are presented as frequency (percentage) and analyzed with the chi-square test to assess the demographic characteristics and clinical pathological data of participants. Comprehensive analyses and data visualization were conducted using Python Version 3.10.6 and R Version 4.5.1. Statistical significance was set at a *P-*value threshold of less than 0.05 (two-sided).

## Results

### Basic characteristics of patients

**FIGURE 1:**
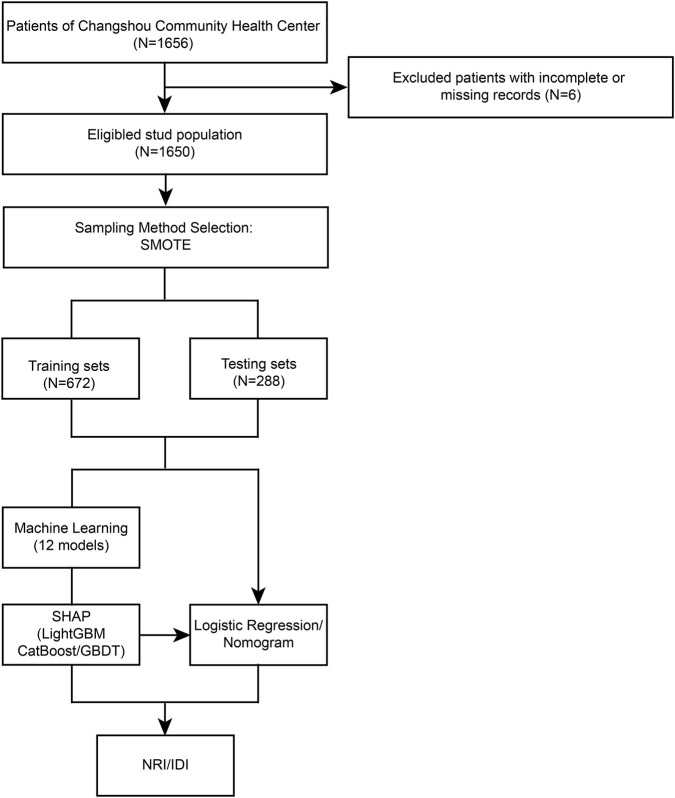
Sample selection flowchart from Changshou Community Health Center and workflow of the whole study.

**FIGURE 2:**
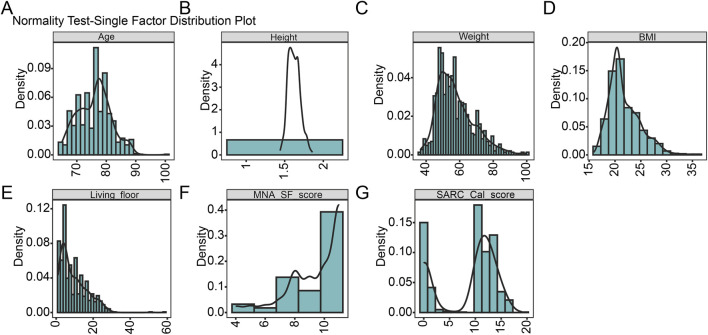
Density distribution and normality assessment of continuous variables using the Kolmogorov-Smirnov test. **(A-G)** Density plots illustrating the distribution of continuous variables included in the analysis. The Kolmogorov-Smirnov test was applied to assess normality for each variable. Abbreviations: BMI, Body Mass Index; MNA-SF, Mini Nutritional Assessment–Short Form; SARC-CalF, SARC-Calcium Score.

A total of 1,656 patients from Changshou Community Health Center were initially enrolled in this study. After excluding six patients due to incomplete or missing medical records, 1,650 eligible participants were retained and balanced by SMOTE method. The patient selection process was illustrated in Figure 1. The cohort was randomly partitioned into a training set (N = 672) and a testing set (N = 288). The normality of continuous variables was assessed using the Kolmogorov-Smirnov test, with results detailed in Table 1. All tested variables—including age, height, weight, BMI, living floor, MNA-SF score, and SARC-Cal score—showed significant deviations from normality (all p < 0.001), indicating non-parametric distributions. The density distributions of above seven variables are visually presented in Figure 2.

**TABLE 1:** Normality assessment of continuous variables by Kolmogorov-Smirnov test.

Variable name	Kolmogorov-Smirnov	P value
Age	0.0949813687644587	7.60796118633877e-23
Weight	0.106947142247959	2.62883408495937e-29
BMI	0.129964457705953	4.76202998616924e-44
Height	0.0561263656375865	1.55455574987522e-07
Living_floor	0.14997477124774	2.4483685758198e-59
MNA_SF_score	0.227017102010177	1.12088507831756e-139
SARC_Cal_score	0.291848507172742	1.06347361153157e-233

The baseline characteristics of participants in the training and testing cohorts were summarized in Tables 2, 3, respectively. Overall, the two cohorts exhibited broadly comparable distributions across key demographic and clinical profiles, supporting the robustness of the subsequent modeling approach.

**TABLE 2:** Baseline characteristics of participants in training cohort. Significant values are in [bold].

Variable	Levels	N	Overall	Non-sarcopenia	Sarcopenia	p-value
			N = 672	N = 341	N = 331	
Age, median (p25 - p75)	​	672	77.00 (72.00–79.00)	72.00 (69.00–76.00)	79.00 (77.00–81.00)	**<0.001**
Height, median (p25 - p75)	​	672	1.62 (1.57–1.68)	1.64 (1.59–1.71)	1.60 (1.55–1.65)	**<0.001**
Weight, median (p25 - p75)	​	672	55.65 (49.62–63.70)	63.50 (57.30–71.00)	50.09 (47.59–54.10)	**<0.001**
BMI, median (p25 - p75)	​	672	21.01 (19.97–23.56)	23.54 (22.01–25.33)	20.13 (19.05–20.58)	**<0.001**
Living_floor, median (p25 - p75)	​	672	7.50 (4.00–14.00)	9.00 (5.00–17.00)	6.00 (4.00–13.00)	**<0.001**
MNA_SF_score, median (p25 - p75)	​	672	10.00 (8.00–11.00)	11.00 (11.00–11.00)	8.00 (8.00–10.00)	**<0.001**
SARC_Cal_score, median (p25 - p75)	​	672	11.00 (1.00–13.00)	1.00 (0.00–10.00)	13.00 (12.00–14.00)	**<0.001**
Gender, n (p%)	​	672	​	​	​	**<0.001**
​	Male	​	400.00 (59.52%)	150.00 (43.99%)	250.00 (75.53%)	​
​	Female	​	272.00 (40.48%)	191.00 (56.01%)	81.00 (24.47%)	​
Marriage, n (p%)	​	672	​	​	​	**<0.001**
​	Unmarried	​	3.00 (0.45%)	3.00 (0.88%)	0.00 (0.00%)	​
​	Married	​	551.00 (81.99%)	293.00 (85.92%)	258.00 (77.95%)	​
​	Divorced	​	71.00 (10.57%)	9.00 (2.64%)	62.00 (18.73%)	​
​	Widowed	​	46.00 (6.85%)	35.00 (10.26%)	11.00 (3.32%)	​
​	Not specified	​	1.00 (0.15%)	1.00 (0.29%)	0.00 (0.00%)	​
Education, n (p%)	​	672	​	​	​	**<0.001**
​	Primary school or below	​	19.00 (2.83%)	5.00 (1.47%)	14.00 (4.23%)	​
​	Junior high school	​	199.00 (29.61%)	93.00 (27.27%)	106.00 (32.02%)	​
​	Senior high school or vocational school	​	246.00 (36.61%)	125.00 (36.66%)	121.00 (36.56%)	​
​	Associate degree	​	161.00 (23.96%)	73.00 (21.41%)	88.00 (26.59%)	​
​	Bachelor’s degree or higher	​	47.00 (6.99%)	45.00 (13.20%)	2.00 (0.60%)	​
Living_status, n (p%)	​	672	​	​	​	**<0.001**
​	No detail	​	1.00 (0.15%)	1.00 (0.29%)	0.00 (0.00%)	​
​	Living alone	​	160.00 (23.81%)	35.00 (10.26%)	125.00 (37.76%)	​
​	Living with spouse only	​	431.00 (64.14%)	226.00 (66.28%)	205.00 (61.93%)	​
​	Living with spouse and children	​	58.00 (8.63%)	57.00 (16.72%)	1.00 (0.30%)	​
​	Living with children only	​	21.00 (3.13%)	21.00 (6.16%)	0.00 (0.00%)	​
​	Living with others	​	1.00 (0.15%)	1.00 (0.29%)	0.00 (0.00%)	​
Hypertension, n (p%)	​	672	​	​	​	**<0.001**
​	No	​	479.00 (71.28%)	183.00 (53.67%)	296.00 (89.43%)	​
​	Yes	​	193.00 (28.72%)	158.00 (46.33%)	35.00 (10.57%)	​
Diabetes, n (p%)	​	672	​	​	​	0.885
​	No	​	563.00 (83.78%)	285.00 (83.58%)	278.00 (83.99%)	​
​	Yes	​	109.00 (16.22%)	56.00 (16.42%)	53.00 (16.01%)	​
COPD, n (p%)	​	672	​	​	​	**0.027**
​	No	​	667.00 (99.26%)	336.00 (98.53%)	331.00 (100.00%)	​
​	Yes	​	5.00 (0.74%)	5.00 (1.47%)	0.00 (0.00%)	​
Hyperuricemia, n (p%)	​	672	​	​	​	**0.028**
​	No	​	652.00 (97.02%)	326.00 (95.60%)	326.00 (98.49%)	​
​	Yes	​	20.00 (2.98%)	15.00 (4.40%)	5.00 (1.51%)	​
Osteoarthritis, n (p%)	​	672	​	​	​	**<0.001**
​	No	​	630.00 (93.75%)	299.00 (87.68%)	331.00 (100.00%)	​
​	Yes	​	42.00 (6.25%)	42.00 (12.32%)	0.00 (0.00%)	​
Osteoporosis, n (p%)	​	672	​	​	​	**<0.001**
​	No	​	645.00 (95.98%)	314.00 (92.08%)	331.00 (100.00%)	​
​	Yes	​	27.00 (4.02%)	27.00 (7.92%)	0.00 (0.00%)	​
Tumor, n (p%)	​	672	​	​	​	**0.005**
​	No	​	664.00 (98.81%)	333.00 (97.65%)	331.00 (100.00%)	​
​	Yes	​	8.00 (1.19%)	8.00 (2.35%)	0.00 (0.00%)	​
CKD, n (p%)	​	672	​	​	​	**0.002**
​	No	​	662.00 (98.51%)	331.00 (97.07%)	331.00 (100.00%)	​
​	Yes	​	10.00 (1.49%)	10.00 (2.93%)	0.00 (0.00%)	​
Other_disease, n (p%)	​	672	​	​	​	**<0.001**
​	No	​	650.00 (96.73%)	320.00 (93.84%)	330.00 (99.70%)	​
​	Yes	​	22.00 (3.27%)	21.00 (6.16%)	1.00 (0.30%)	​
Chronic_Disease_number, n (p%)	​	672	​	​	​	**<0.001**
​	0	​	208.00 (30.95%)	94.00 (27.57%)	114.00 (34.44%)	​
​	1	​	290.00 (43.15%)	125.00 (36.66%)	165.00 (49.85%)	​
​	2	​	110.00 (16.37%)	77.00 (22.58%)	33.00 (9.97%)	​
​	3	​	53.00 (7.89%)	34.00 (9.97%)	19.00 (5.74%)	​
​	4	​	8.00 (1.19%)	8.00 (2.35%)	0.00 (0.00%)	​
​	5	​	1.00 (0.15%)	1.00 (0.29%)	0.00 (0.00%)	​
​	6	​	2.00 (0.30%)	2.00 (0.59%)	0.00 (0.00%)	​
Pension, n (p%)	​	672	​	​	​	**<0.001**
​	<5,000	​	237.00 (35.27%)	91.00 (26.69%)	146.00 (44.11%)	​
​	5,000–9,999	​	413.00 (61.46%)	239.00 (70.09%)	174.00 (52.57%)	​
​	10,000–14,999	​	22.00 (3.27%)	11.00 (3.23%)	11.00 (3.32%)	​
Drug, n (p%)	​	672	​	​	​	**<0.001**
​	None	​	145.00 (21.58%)	88.00 (25.81%)	57.00 (17.22%)	​
​	One	​	239.00 (35.57%)	98.00 (28.74%)	141.00 (42.60%)	​
​	Two	​	161.00 (23.96%)	60.00 (17.60%)	101.00 (30.51%)	​
​	Three or more	​	127.00 (18.90%)	95.00 (27.86%)	32.00 (9.67%)	​
Sleep_duration, n (p%)	​	672	​	​	​	**<0.001**
​	<5 h	​	267.00 (39.73%)	56.00 (16.42%)	211.00 (63.75%)	​
​	≥5 h	​	405.00 (60.27%)	285.00 (83.58%)	120.00 (36.25%)	​
Outdoor_activity_time, n (p%)	​	672	​	​	​	**<0.001**
​	<1 h	​	429.00 (63.84%)	128.00 (37.54%)	301.00 (90.94%)	​
​	1–3 h	​	233.00 (34.67%)	203.00 (59.53%)	30.00 (9.06%)	​
​	>3 h	​	10.00 (1.49%)	10.00 (2.93%)	0.00 (0.00%)	​
Living_house, n (p%)	​	672	​	​	​	**<0.001**
​	Building with elevator	​	597.00 (88.84%)	280.00 (82.11%)	317.00 (95.77%)	​
​	Building without elevator	​	75.00 (11.16%)	61.00 (17.89%)	14.00 (4.23%)	​
Living_area, n (p%)	​	672	​	​	​	**<0.001**
​	50–100 m^2^	​	349.00 (51.93%)	139.00 (40.76%)	210.00 (63.44%)	​
​	100–150 m^2^	​	250.00 (37.20%)	167.00 (48.97%)	83.00 (25.08%)	​
​	>150 m^2^	​	53.00 (7.89%)	21.00 (6.16%)	32.00 (9.67%)	​
​	<50 m^2^	​	20.00 (2.98%)	14.00 (4.11%)	6.00 (1.81%)	​
Smoking, n (p%)	​	672	​	​	​	**<0.001**
​	Yes	​	133.00 (19.79%)	33.00 (9.68%)	100.00 (30.21%)	​
​	No	​	539.00 (80.21%)	308.00 (90.32%)	231.00 (69.79%)	​
Drinking, n (p%)	​	672	​	​	​	0.327
​	Yes	​	79.00 (11.76%)	36.00 (10.56%)	43.00 (12.99%)	​
​	No	​	593.00 (88.24%)	305.00 (89.44%)	288.00 (87.01%)	​
Coffee_tea, n (p%)	​	672	​	​	​	**<0.001**
​	Yes	​	238.00 (35.42%)	94.00 (27.57%)	144.00 (43.50%)	​
​	No	​	434.00 (64.58%)	247.00 (72.43%)	187.00 (56.50%)	​
Physical_exercise, n (p%)	​	672	​	​	​	**0.004**
​	Yes	​	426.00 (63.39%)	234.00 (68.62%)	192.00 (58.01%)	​
​	No	​	246.00 (36.61%)	107.00 (31.38%)	139.00 (41.99%)	​
Teeth, n (p%)	​	672	​	​	​	**<0.001**
​	Tooth loss	​	278.00 (41.37%)	97.00 (28.45%)	181.00 (54.68%)	​
​	Denture use	​	296.00 (44.05%)	161.00 (47.21%)	135.00 (40.79%)	​
​	Functional dentition	​	98.00 (14.58%)	83.00 (24.34%)	15.00 (4.53%)	​
GLIM_Phenotypic criteria, n (p%)	​	672	​	​	​	**<0.001**
​	Non-volitional weight loss	​	129.00 (19.20%)	18.00 (5.28%)	111.00 (33.53%)	​
​	Low BMI	​	116.00 (17.26%)	15.00 (4.40%)	101.00 (30.51%)	​
​	Reduced muscle mass	​	90.00 (13.39%)	4.00 (1.17%)	86.00 (25.98%)	​
​	None	​	337.00 (50.15%)	304.00 (89.15%)	33.00 (9.97%)	​
GLIM_Etiologic criteria, n (p%)	​	672	​	​	​	**<0.001**
​	Reduced food intake	​	106.00 (15.77%)	13.00 (3.81%)	93.00 (28.10%)	​
​	Inflammation/Disease burden	​	160.00 (23.81%)	34.00 (9.97%)	126.00 (38.07%)	​
​	None	​	406.00 (60.42%)	294.00 (86.22%)	112.00 (33.84%)	​
GLIM-malnutrition, n (p%)	​	672	​	​	​	**<0.001**
​	Moderate malnutrition	​	184.00 (27.38%)	9.00 (2.64%)	175.00 (52.87%)	​
​	Severe malnutrition	​	488.00 (72.62%)	332.00 (97.36%)	156.00 (47.13%)	​

**TABLE 3:** Baseline characteristics of participants in testing cohort. Significant values are in [bold].

Variable	Levels	N	Overall	Non-sarcopenia	Sarcopenia	p-value
			N = 288	N = 139	N = 149	
Age, median (p25 - p75)	​	288	77.00 (72.00–80.00)	73.00 (69.00–76.00)	79.00 (77.00–81.00)	**<0.001**
Weight, median (p25 - p75)	​	288	55.21 (48.73–63.70)	64.00 (56.70–71.90)	49.12 (46.53–54.08)	**<0.001**
Height, median (p25 - p75)	​	288	1.61 (1.55–1.67)	1.65 (1.59–1.71)	1.59 (1.54–1.65)	**<0.001**
BMI, median (p25 - p75)	​	288	20.71 (19.60–23.50)	23.56 (21.52–26.05)	19.91 (18.73–20.43)	**<0.001**
Living_floor, median (p25 - p75)	​	288	7.00 (4.00–12.00)	10.00 (5.00–16.00)	5.00 (3.00–10.00)	**<0.001**
MNA_SF_score, median (p25 - p75)	​	288	10.00 (8.00–11.00)	11.00 (11.00–11.00)	9.00 (8.00–10.00)	**<0.001**
SARC_Cal_score, median (p25 - p75)	​	288	11.00 (1.00–13.00)	1.00 (0.00–10.00)	12.00 (12.00–14.00)	**<0.001**
Gender, n (p%)	​	288	​	​	​	**<0.001**
​	Male	​	177.00 (61.46%)	64.00 (46.04%)	113.00 (75.84%)	​
​	Female	​	111.00 (38.54%)	75.00 (53.96%)	36.00 (24.16%)	​
Marriage, n (p%)	​	288	​	​	​	**<0.001**
​	Unmarried	​	1.00 (0.35%)	1.00 (0.72%)	0.00 (0.00%)	​
​	Married	​	244.00 (84.72%)	119.00 (85.61%)	125.00 (83.89%)	​
​	Divorced	​	26.00 (9.03%)	3.00 (2.16%)	23.00 (15.44%)	​
​	Widowed	​	17.00 (5.90%)	16.00 (11.51%)	1.00 (0.67%)	​
Education, n (p%)	​	288	​	​	​	**<0.001**
​	Primary school or below	​	14.00 (4.86%)	4.00 (2.88%)	10.00 (6.71%)	​
​	Junior high school	​	87.00 (30.21%)	33.00 (23.74%)	54.00 (36.24%)	​
​	Senior high school or vocational school	​	94.00 (32.64%)	49.00 (35.25%)	45.00 (30.20%)	​
​	Associate degree	​	81.00 (28.13%)	41.00 (29.50%)	40.00 (26.85%)	​
​	Bachelor’s degree or higher	​	12.00 (4.17%)	12.00 (8.63%)	0.00 (0.00%)	​
Living_status, n (p%)	​	288	​	​	​	**<0.001**
​	No detail	​	65.00 (22.57%)	19.00 (13.67%)	46.00 (30.87%)	​
​	Living alone	​	194.00 (67.36%)	92.00 (66.19%)	102.00 (68.46%)	​
​	Living with spouse only	​	23.00 (7.99%)	22.00 (15.83%)	1.00 (0.67%)	​
​	Living with spouse and children	​	6.00 (2.08%)	6.00 (4.32%)	0.00 (0.00%)	​
Hypertension, n (p%)	​	288	​	​	​	**<0.001**
​	No	​	204.00 (70.83%)	66.00 (47.48%)	138.00 (92.62%)	​
​	Yes	​	84.00 (29.17%)	73.00 (52.52%)	11.00 (7.38%)	​
Diabetes, n (p%)	​	288	​	​	​	0.818
​	No	​	250.00 (86.81%)	120.00 (86.33%)	130.00 (87.25%)	​
​	Yes	​	38.00 (13.19%)	19.00 (13.67%)	19.00 (12.75%)	​
COPD, n (p%)	​	288	​	​	​	0.961
​	No	​	286.00 (99.31%)	138.00 (99.28%)	148.00 (99.33%)	​
​	Yes	​	2.00 (0.69%)	1.00 (0.72%)	1.00 (0.67%)	​
Hyperuricemia, n (p%)	​	288	​	​	​	0.327
​	No	​	275.00 (95.49%)	131.00 (94.24%)	144.00 (96.64%)	​
​	Yes	​	13.00 (4.51%)	8.00 (5.76%)	5.00 (3.36%)	​
Osteoarthritis, n (p%)	​	288	​	​	​	**0.002**
​	No	​	276.00 (95.83%)	128.00 (92.09%)	148.00 (99.33%)	​
​	Yes	​	12.00 (4.17%)	11.00 (7.91%)	1.00 (0.67%)	​
Osteoporosis, n (p%)	​	288	​	​	​	**0.004**
​	No	​	277.00 (96.18%)	129.00 (92.81%)	148.00 (99.33%)	​
​	Yes	​	11.00 (3.82%)	10.00 (7.19%)	1.00 (0.67%)	​
Tumor, n (p%)	​	288	​	​	​	**0.037**
​	No	​	284.00 (98.61%)	135.00 (97.12%)	149.00 (100.00%)	​
​	Yes	​	4.00 (1.39%)	4.00 (2.88%)	0.00 (0.00%)	​
CKD, n (p%)	​	288	​	​	​	0.071
​	No	​	285.00 (98.96%)	136.00 (97.84%)	149.00 (100.00%)	​
​	Yes	​	3.00 (1.04%)	3.00 (2.16%)	0.00 (0.00%)	​
Other_disease, n (p%)	​	288	​	​	​	**<0.001**
​	No	​	278.00 (96.53%)	129.00 (92.81%)	149.00 (100.00%)	​
​	Yes	​	10.00 (3.47%)	10.00 (7.19%)	0.00 (0.00%)	​
Chronic_Disease_number, n (p%)	​	288	​	​	​	**0.004**
​	0	​	106.00 (36.81%)	38.00 (27.34%)	68.00 (45.64%)	​
​	1	​	114.00 (39.58%)	57.00 (41.01%)	57.00 (38.26%)	​
​	2	​	41.00 (14.24%)	28.00 (20.14%)	13.00 (8.72%)	​
​	3	​	20.00 (6.94%)	10.00 (7.19%)	10.00 (6.71%)	​
​	4	​	6.00 (2.08%)	5.00 (3.60%)	1.00 (0.67%)	​
​	6	​	1.00 (0.35%)	1.00 (0.72%)	0.00 (0.00%)	​
Pension, n (p%)	​	288	​	​	​	**<0.001**
​	<5,000	​	110.00 (38.19%)	39.00 (28.06%)	71.00 (47.65%)	​
​	5,000–9,999	​	166.00 (57.64%)	90.00 (64.75%)	76.00 (51.01%)	​
​	10,000–14,999	​	12.00 (4.17%)	10.00 (7.19%)	2.00 (1.34%)	​
Drug, n (p%)	​	288	​	​	​	**<0.001**
​	None	​	69.00 (23.96%)	33.00 (23.74%)	36.00 (24.16%)	​
​	One	​	94.00 (32.64%)	32.00 (23.02%)	62.00 (41.61%)	​
​	Two	​	73.00 (25.35%)	29.00 (20.86%)	44.00 (29.53%)	​
​	Three or more	​	52.00 (18.06%)	45.00 (32.37%)	7.00 (4.70%)	​
Sleep_duration, n (p%)	​	288	​	​	​	**<0.001**
​	<5 h	​	114.00 (39.58%)	24.00 (17.27%)	90.00 (60.40%)	​
​	≥5 h	​	174.00 (60.42%)	115.00 (82.73%)	59.00 (39.60%)	​
Outdoor_activity_time, n (p%)	​	288	​	​	​	**<0.001**
​	<1 h	​	182.00 (63.19%)	42.00 (30.22%)	140.00 (93.96%)	​
​	1–3 h	​	100.00 (34.72%)	91.00 (65.47%)	9.00 (6.04%)	​
​	>3 h	​	6.00 (2.08%)	6.00 (4.32%)	0.00 (0.00%)	​
Living_house, n (p%)	​	288	​	​	​	**<0.001**
​	Building with elevator	​	249.00 (86.46%)	108.00 (77.70%)	141.00 (94.63%)	​
​	Building without elevator	​	39.00 (13.54%)	31.00 (22.30%)	8.00 (5.37%)	​
Living_area, n (p%)	​	288	​	​	​	**<0.001**
​	50–100 m^2^	​	158.00 (54.86%)	58.00 (41.73%)	100.00 (67.11%)	​
​	100–150 m^2^	​	94.00 (32.64%)	63.00 (45.32%)	31.00 (20.81%)	​
​	>150 m^2^	​	27.00 (9.38%)	11.00 (7.91%)	16.00 (10.74%)	​
​	<50 m^2^	​	9.00 (3.13%)	7.00 (5.04%)	2.00 (1.34%)	​
Smoking, n (p%)	​	288	​	​	​	**<0.001**
​	Yes	​	64.00 (22.22%)	19.00 (13.67%)	45.00 (30.20%)	​
​	No	​	224.00 (77.78%)	120.00 (86.33%)	104.00 (69.80%)	​
Drinking, n (p%)	​	288	​	​	​	0.119
​	Yes	​	36.00 (12.50%)	13.00 (9.35%)	23.00 (15.44%)	​
​	No	​	252.00 (87.50%)	126.00 (90.65%)	126.00 (84.56%)	​
Coffee_tea, n (p%)	​	288	​	​	​	**0.004**
​	Yes	​	116.00 (40.28%)	44.00 (31.65%)	72.00 (48.32%)	​
​	No	​	172.00 (59.72%)	95.00 (68.35%)	77.00 (51.68%)	​
Physical_exercise, n (p%)	​	288	​	​	​	**<0.001**
​	Yes	​	191.00 (66.32%)	110.00 (79.14%)	81.00 (54.36%)	​
​	No	​	97.00 (33.68%)	29.00 (20.86%)	68.00 (45.64%)	​
Teeth, n (p%)	​	288	​	​	​	**<0.001**
​	Tooth loss	​	119.00 (41.32%)	36.00 (25.90%)	83.00 (55.70%)	​
​	Denture use	​	133.00 (46.18%)	75.00 (53.96%)	58.00 (38.93%)	​
​	Functional dentition	​	36.00 (12.50%)	28.00 (20.14%)	8.00 (5.37%)	​
GLIM_Phenotypic criteria, n (p%)	​	288	​	​	​	**<0.001**
​	Non-volitional weight loss	​	47.00 (16.32%)	7.00 (5.04%)	40.00 (26.85%)	​
​	Low BMI	​	61.00 (21.18%)	5.00 (3.60%)	56.00 (37.58%)	​
​	Reduced muscle mass	​	35.00 (12.15%)	0.00 (0.00%)	35.00 (23.49%)	​
​	None	​	145.00 (50.35%)	127.00 (91.37%)	18.00 (12.08%)	​
GLIM_Etiologic criteria, n (p%)	​	288	​	​	​	**<0.001**
​	Reduced food intake	​	42.00 (14.58%)	7.00 (5.04%)	35.00 (23.49%)	​
​	Inflammation/Disease burden	​	65.00 (22.57%)	11.00 (7.91%)	54.00 (36.24%)	​
​	None	​	181.00 (62.85%)	121.00 (87.05%)	60.00 (40.27%)	​
GLIM-malnutrition, n (p%)	​	288	​	​	​	**<0.001**
​	Moderate malnutrition	​	77.00 (26.74%)	3.00 (2.16%)	74.00 (49.66%)	​
​	Severe malnutrition	​	211.00 (73.26%)	136.00 (97.84%)	75.00 (50.34%)	​

In the training cohort (N = 672), 331 participants (49.3%) were diagnosed with sarcopenia, compared to 149 (51.7%) in the testing cohort (N = 288). Significant differences between the sarcopenic and non-sarcopenic groups were observed in both cohorts for core anthropometric measures—including age, height, weight, and body mass index (BMI)—as well as for functional and nutritional assessments such as the MNA-SF score and SARC-Cal score (all p < 0.001). Numerous socio-demographic, comorbidity, and lifestyle variables (e.g., gender, marriage, living status, pension level, physical activity, and several chronic diseases) also showed statistically significant differences between groups (p < 0.05). In contrast, the prevalence of certain conditions, including diabetes mellitus and hyperuricemia, did not differ significantly between the sarcopenic and non-sarcopenic groups in either cohort (p > 0.05). Similarly, the variable “Drinking” showed no significant difference in either cohort (training: p = 0.327; testing: p = 0.119), indicating balanced baseline characteristics for these specific parameters. The overall similarity between the training and testing sets enhanced the reliability and generalizability of the model developed in this study.

### Sarcopenia prediction model using machine learning methods

**FIGURE 3:**
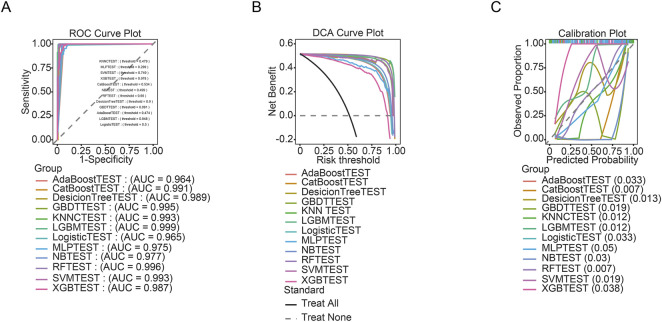
Development and validation of machine learning predictive models. **(A)** Receiver Operating Characteristic (ROC) curves for the twelve evaluated algorithms on the independent testing set. The corresponding Area Under the Curve (AUC) values are displayed in the legend. Multiple models. **(B)** Decision Curve Analysis (DCA) for the machine learning models. The y-axis represents net benefit, and the x-axis represents the risk threshold. **(C)** Calibration plots assessing the agreement between predicted probabilities and observed outcomes. A perfect calibration would follow the 45-degree dotted line. The mean absolute error (values in parentheses) for each model indicates excellent calibration for the top-performing models (e.g., CatBoost TEST: 0.007).

Multiple machine learning models were developed to predict risk factors associated with the occurrence of sarcopenia. Based on their superior discriminative performance, CatBoost, LightGBM, and Gradient Boosting Decision Tree (GBDT) were selected for final evaluation. All three models achieved exceptional area under the receiver operating characteristic curve (AUROC) values above 0.995, with CatBoost reaching a near-optimal score of 0.999 (Table 4). This high discriminative ability was visually reflected in their ROC curves, which occupied the upper-left corner of the plot, confirming excellent diagnostic accuracy (Figure 3A). Beyond AUROC, these models exhibited balanced performance across other key metrics: CatBoost and LightGBM attained perfect sensitivity (Recall = 1.000), while all three maintained high precision (>0.967), F1-scores (>0.976), and substantial Matthews correlation coefficients (>0.951). Decision curve analysis (Figure 3B) demonstrated that using any of the three models yielded substantially greater net clinical benefit across a wide range of threshold probabilities compared to “treat-all” or “treat-none” strategies, underscoring their potential utility for risk stratification. Furthermore, calibration curves for all models closely followed the ideal diagonal (Figure 3C), indicating well-calibrated and reliable probability estimates. Together, the near-perfect discrimination, meaningful net benefit, and strong calibration support the robustness of these ensemble models. This suggests that the models hold potential clinical utility for guiding decision-making in sarcopenia risk intervention.

**TABLE 4:** Evaluation metrics of different machine learning algorithms.

Model name	Accuracy	Prevalence	Recall	F1-score	MCC	AUROC	Precision	Specificity	FNR	FPR
LogisticTEST	0.96875	0.5173611111111112	0.9798657718120806	0.9700996677740865	0.9375834818664643	0.9768721935203515	0.9605263157894737	0.9568345323741008	0.020134228187919462	0.04316546762589928
LGBMTEST	0.9895833333333334	0.5173611111111112	1	0.9900332225913622	0.979339777141342	0.9962821688957558	0.9802631578947368	0.9784172661870504	0	0.02158273381294964
AdaBoostTEST	0.9652777777777778	0.5173611111111112	0.9865771812080537	0.9671052631578947	0.9311838510995959	0.9645116121867607	0.9483870967741935	0.9424460431654677	0.013422818791946308	0.05755395683453238
GBDTTEST	0.9756944444444444	0.5173611111111112	0.9731543624161074	0.9764309764309763	0.951364942434089	0.9954130655207377	0.9797297297297297	0.9784172661870504	0.026845637583892617	0.02158273381294964
DesicionTreeTEST	0.9652777777777778	0.5173611111111112	0.9932885906040269	0.9673202614379084	0.9317853103796249	0.9642701945825889	0.9426751592356688	0.935251798561151	0.006711409395973154	0.06474820143884892
RFTEST	0.9652777777777778	0.5173611111111112	0.9932885906040269	0.9673202614379084	0.9317853103796249	0.9926609048331805	0.9426751592356688	0.935251798561151	0.006711409395973154	0.06474820143884892
NBTEST	0.9097222222222222	0.5173611111111112	1	0.9197530864197531	0.8319666765403448	0.9870117328955628	0.8514285714285714	0.8129496402877698	0	0.18705035971223022
CatBoostTEST	0.9826388888888888	0.5173611111111112	1	0.9834983498349835	0.9657790334037258	0.9992274636666505	0.9675324675324676	0.9640287769784173	0	0.03597122302158273
XGBTEST	0.9895833333333334	0.5173611111111112	1	0.9900332225913622	0.979339777141342	0.9931437400415238	0.9802631578947368	0.9784172661870504	0	0.02158273381294964
SVMTEST	0.9375	0.5173611111111112	1	0.9430379746835443	0.8812927221435178	0.9746028680411376	0.8922155688622755	0.8705035971223022	0	0.12949640287769784
MLPTEST	0.9930555555555556	0.5173611111111112	1	0.9933333333333334	0.9861830731130972	0.9907778475206411	0.9867549668874173	0.9856115107913669	0	0.014388489208633094
KNNCTEST	0.9652777777777778	0.5173611111111112	1	0.9675324675324676	0.9325712596562837	0.9890637825310222	0.9371069182389937	0.9280575539568345	0	0.07194244604316546

### Model calibration and validation

**FIGURE 4:**
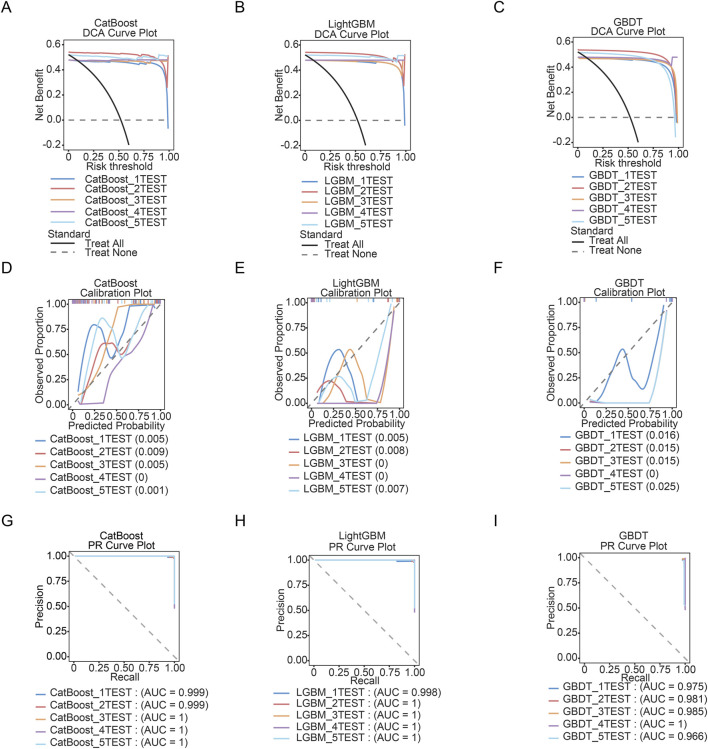
CatBoost, LightGBM and GBDT models optimized by 5-fold cross-validation. **(A)** Decision Curve Analysis (DCA) for CatBoost model. The y-axis represents net benefit, and the x-axis represents the risk threshold. **(B)** Decision Curve Analysis (DCA) for LightGBM model. The y-axis represents net benefit, and the x-axis represents the risk threshold. **(C)** Decision Curve Analysis (DCA) for GBDT model. The y-axis represents net benefit, and the x-axis represents the risk threshold. **(D)** Calibration plots for CatBoost model. **(E)** Calibration plots for LightGBM model. **(F)** Calibration plots for GBDT model. **(G)** PR Curve plot for CatBoost model. **(H)** PR Curve plot for LightGBM model. **(I)** PR Curve plot for GBDT model.

The clinical utility and robustness of the top-performing models—CatBoost, LightGBM, and GBDT—were further assessed through decision curve analysis (DCA), calibration plots, and precision-recall (PR) curves, as summarized in Figure 4. These evaluations were conducted across all five test folds of the 5-fold cross-validation, with corresponding accuracy results provided in Table 5.

**TABLE 5:** Results of 5-fold cross-validation in CatBoost, LightGBM and GBDT models.

Accuracy	1 TEST	2 TEST	3 TEST	4 TEST	5 TEST	Mean_scores
CatBoostTEST	0.984375	0.984375	0.989583333	1	0.973958333	0.986458333
LGBMTEST	0.989583333	0.989583333	0.989583333	1	0.989583333	0.991666667
GBDTTEST	0.979166667	0.984375	0.984375	0.994791667	0.973958333	0.983333333

As shown in Figures 4A–C, the DCA indicated that all three models yielded a higher net benefit over a wide range of risk thresholds compared to the “treat all” or “treat none” strategies, supporting their potential clinical value for identifying individuals at risk of sarcopenia. The calibration plots showed close agreement between predicted probabilities and observed outcomes, with low mean absolute errors (e.g., 0.001) across test folds, confirming excellent calibration reliability (Figures 4D–F). The PR curves further affirmed the models’ strong performance, with the area under the PR curve (AUC-PR) approaching or reaching 1.0 in several folds (Figures 4G–I). For example, CatBoost and LightGBM attained perfect AUC-PR values (AUC = 1) in at least three of the five folds, while GBDT also maintained consistently high AUC-PR scores, reflecting a robust balance between precision and recall despite class imbalance.

Overall, these results align with the high cross-validation accuracy reported in Table 5 (mean scores >0.98), confirming that the models are not only accurate but also well-calibrated and clinically applicable. LightGBM, which achieved the highest mean accuracy (0.992), also demonstrated the most consistent and superior performance in both calibration and PR analyses.

### Interpretation of the catboost, LightGBM and GBDT models

**FIGURE 5:**
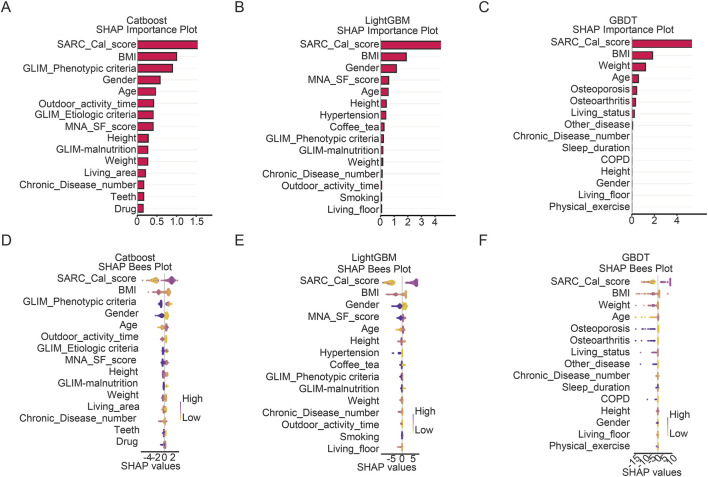
Feature importance and SHAP value distributions for the top15 predictors using CatBoost, LightGBM and GBDT models. **(A)** Mean absolute SHAP value-based feature importance plot for the CatBoost model. **(B)** Mean absolute SHAP value-based feature importance plot for the LightGBM model. **(C)** Mean absolute SHAP value-based feature importance plot for the GBDT model. **(D)** Beeswarm plot of SHAP values for the CatBoost model. **(E)** Beeswarm plot of SHAP values for the LightGBM model. **(F)** Beeswarm plot of SHAP values for the GBDT model.

Figure 5 illustrated the SHAP analysis results for the three top-performing models- CatBoost, LightGBM, and GBDT-elucidating the contribution of individual features to sarcopenia risk prediction. Figures 5A–C depicted the summary plots of feature importance, quantified by the mean absolute SHAP value, for each respective model. The SARC-CalF score and BMI consistently emerged as the most influential predictors across all models. Other prominent features included weight, Gender, GLIM_Phenotypic criteria, MNA_SF_score, osteoporosis and age, though their relative rankings varied. For example, Gender ranked highly in LightGBM and CatBoost but was less dominant in GBDT, which assigned greater importance to weight.

Figures 5D–F present beeswarm plots that visualize the distribution and directional influence of each feature. Elevated SARC-CalF score, advanced age, male, and lower body weight were consistently associated with increased sarcopenia risk (positive SHAP values). Conversely, elevated MNA_SF_score, higher BMI and female were generally correlated with reduced risk (negative SHAP values). The directional agreement of these key predictors underscores their robustness.

In summary, the SHAP analysis not only confirms the strong effects of known sarcopenia risk factors but also affords model-specific interpretability, revealing both shared and distinct feature contribution patterns among the ensemble methods.

### Comparative clinical evaluation and nomogram development for sarcopenia risk prediction

**FIGURE 6:**
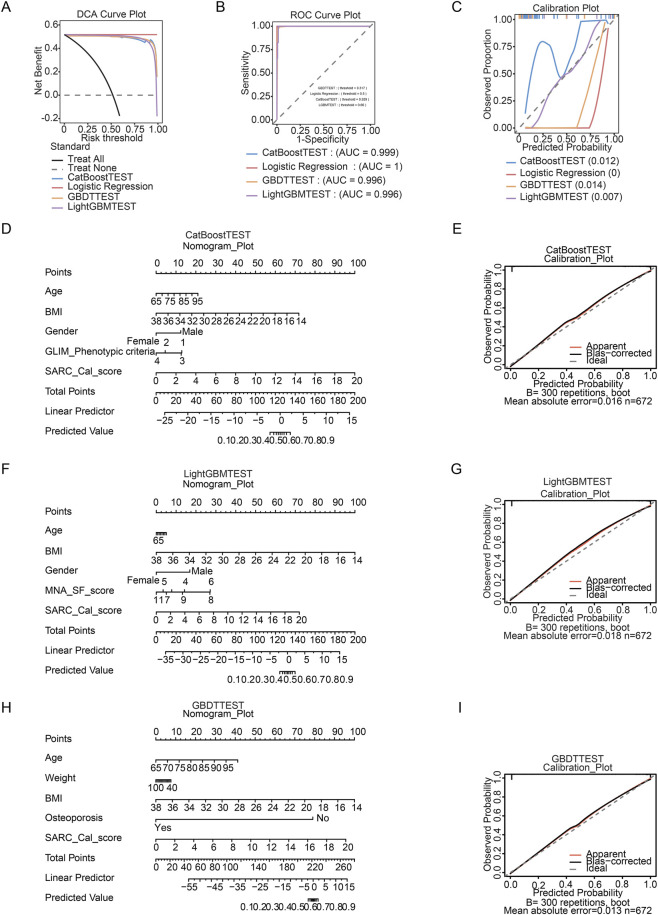
Comprehensive validation, calibration, and nomogram presentation of machine learning models for sarcopenia risk prediction. **(A)** Decision curve analysis (DCA) of the prediction models. The net benefit of each model is plotted against the risk threshold probability. **(B)** Receiver operating characteristic (ROC) curves for the models. **(C)** Calibration curves for the models. **(D)** Nomograms for clinical translation of the CatBoost model TOP5 factors. Note:in terms of GLIM_Phenotypic criteria, 1. represent non-volitional weight loss, 2. represent low BMI, 3. represent reduced muscle mass and 4. represent none. **(E)** Bootstrap-corrected calibration plots (300 repetitions) for the CatBoost model TOP5 factors related Nomogram. **(F)** Nomograms for clinical translation of the LightGBM model TOP5 factors. **(G)** Bootstrap-corrected calibration plots (300 repetitions) for the LightGBM model TOP5 factors related Nomogram. **(H)** Nomograms for clinical translation of the GBDT model TOP5 factors. **(I)** Bootstrap-corrected calibration plots (300 repetitions) for the GBDT model TOP5 factors related Nomogram.

The performance of the final tree-based ensemble models (CatBoost, LightGBM, and GBDT) was evaluated against logistic regression (Table 6; Figure 6). Decision curve analysis (Figure 6A) indicated that all three ensemble models provided greater net clinical benefit across most decision thresholds than both the “treat-all” or “treat-none” strategies and the logistic regression model, underscoring their superior potential for guiding clinical interventions. Although receiver operating characteristic (ROC) curves confirmed high discriminative ability for all classifiers (AUC range: 0.995 to 1.000; Figure 6B), logistic regression failed to identify any positive cases (sensitivity = 0), rendering its F1-score incalculable (Table 6). In contrast, the tree-based models maintained a balanced performance profile: CatBoost and LightGBM achieved perfect sensitivity (1.000) with high precision (>0.973), and all three models demonstrated strong overall accuracy. Furthermore, calibration analysis (Figure 6C) showed that all models were well calibrated, with mean absolute errors below 0.015, confirming the reliability of their predicted probabilities. Together, the demonstrated net clinical benefit, balanced classification performance, and reliable calibration supported the clinical applicability of these ensemble models over traditional logistic regression for this predictive task.

**TABLE 6:** Evaluation metrics of tree-based models versus logistic regression.

Model name	Accuracy	Prevalence	Recall	F1-score	MCC	AUROC	Precision	Specificity	FNR	FPR
LightGBMTEST	0.989583333	0.517361111	1	0.990033223	0.979339777	0.996282169	0.980263158	0.978417266	0	0.021582734
CatBoostTEST	0.986111111	0.517361111	1	0.986754967	0.972538724	0.99917918	0.973856209	0.971223022	0	0.028776978
GBDTTEST	0.982638889	0.517361111	0.973154362	0.983050847	0.96547077	0.995557916	0.993150685	0.992805755	0.026845638	0.007194245
Logistic regression	0.482638889	0.517361111	0	NA	NA	1	NA	1	1	0

Nomograms were constructed from the CatBoost, LightGBM, and Gradient Boosting Decision Tree (GBDT) models (Figures 6D,F,H) to serve as intuitive tools for estimating an individual’s probability of sarcopenia. Each nomogram incorporated a distinct set of predictors selected by the corresponding algorithm: CatBoost used Age, BMI, Gender, GLIM_Phenotypic criteria, and SARC_Cal_score; LightGBM included Age, BMI, Gender, MNA_SF_score, and SARC_Cal_score; and GBDT utilized Age, Weight, BMI, Osteoporosis status, and SARC_Cal_score. The SARC_Cal_score emerged as a major, consistent contributor across all three nomograms, underscoring its central role in risk assessment. Internal validation via bootstrap calibration showed close agreement between predicted and observed outcomes for the CatBoost and LightGBM nomograms (Figures 6E,G), with calibration curves aligning well with the ideal diagonal. This was quantified by low mean absolute error (MAE) values (0.016 for CatBoost and 0.018 for LightGBM); the GBDT model also demonstrated good calibration (MAE = 0.013) (Figure 6I).

In summary, while all tree-based ensembles exhibited near-perfect discrimination, they substantially outperformed logistic regression in recall and net clinical benefit. The resulting nomograms, validated by excellent calibration (all MAE <0.02), represent clinically applicable instruments for individualized sarcopenia risk assessment.

### Comparative assessment of multivariable logistic regression models based on machine learning-derived feature sets

**FIGURE 7:**
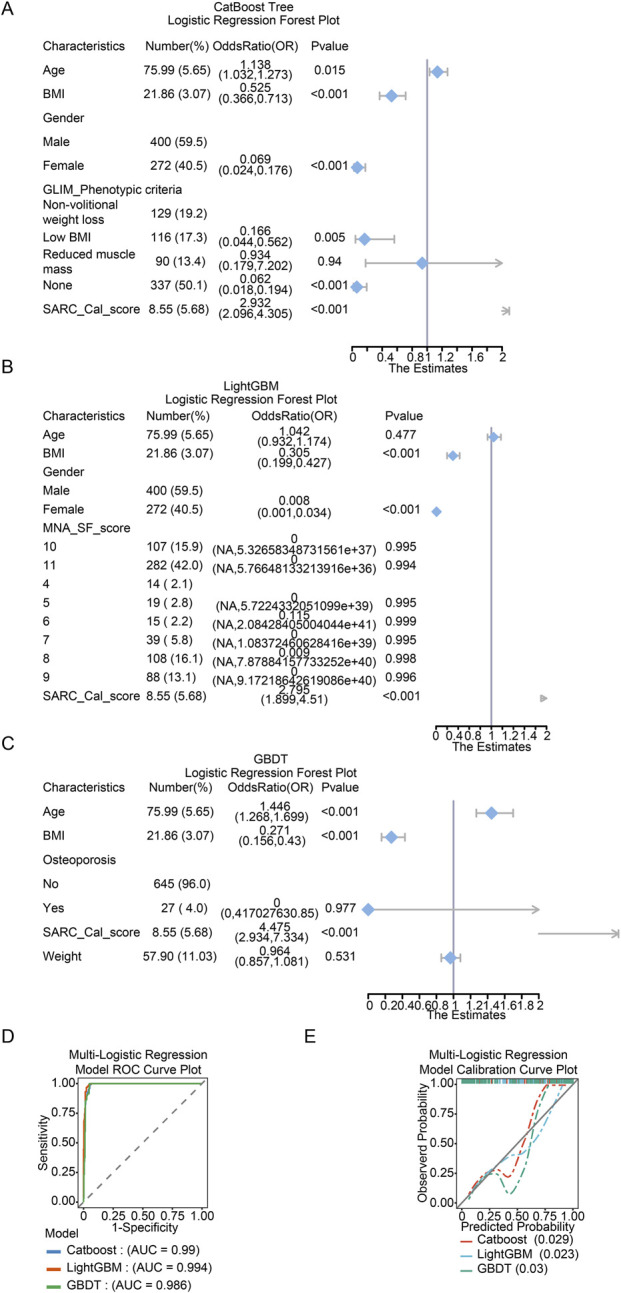
Logistic regression forest plots depicting the sarcopenia prediction models based on features selected by different algorithms. **(A)** Forest plot for the logistic regression model derived from the top5 features of the CatBoost model. **(B)** Forest plot for the logistic regression model derived from the top5 features of the LightGBM model. **(C)** Forest plot for the logistic regression model derived from the top5 features of the GBDT model. **(D)** Receiver Operating Characteristic (ROC) curves for the three multivariable logistic regression models ('CatBoost’, 'LightGBM’, and 'GBDT’). **(E)** Calibration plots assessing the agreement between predicted probabilities and observed outcomes.

To validate the interpretability of the selected tree-based ensemble models, multivariable logistic regression models were constructed using the distinct feature sets identified by CatBoost, LightGBM, and GBDT. Their predictor associations and performance were summarized in Figure 7. Forest plots (Figures 7A–C) depicted the odds ratios (ORs) and 95% confidence intervals for key predictors within each model. The SARC_Cal_score was a consistent, highly significant positive predictor across all models (CatBoost: OR = 2.932 (2.096–4.305), p < 0.001; LightGBM: OR = 2.795 (1.899–4.51), p < 0.001; GBDT: OR = 4.475 (2.934–7.334), p < 0.001). Conversely, BMI was a significant protective factor in both the CatBoost- and LightGBM-derived models (OR = 0.525 (0.366–0.713), p < 0.001; OR = 0.305 (0.199-0.427), p < 0.001). The CatBoost-based model also identified age (OR = 1.138 (1.032–1.273), p = 0.015), female (OR = 0.069 (0.024–0.176), p < 0.001). Moreover, the GBDT-based model highlighted age (OR = 1.446 (1.268–1.699), p < 0.001). Regarding discrimination, receiver operating characteristic (ROC) curves for all three logistic models demonstrated excellent performance (Figure 7D). The LightGBM-based model achieved the highest area under the curve (AUC = 0.994), followed by the CatBoost-based (AUC = 0.990) and GBDT-based models (AUC = 0.986). The calibration curves in Figure 7E further demonstrated close agreement between predicted probabilities and observed outcomes, supported by low mean absolute errors (MAE range: 0.023–0.030).

The predictive performance of the logistic regression models derived from different tree-based ensembles was further assessed using net reclassification improvement (NRI) and integrated discrimination improvement (IDI) metrics (Tables 7–9). Statistically significant differences in reclassification accuracy were observed between the model pairs.

**TABLE 7:** NRI and IDI metrics for the CatBoost TEST Factors model and LightGBM TEST Factors model.

Row name	Value	SE	Z_value	CI_95_lower	CI_95_upper	P value
NRI	1.1004	0.0622	17.6955	0.9785	1.2223	<0.0001
IDI	0.041	0.0089	4.6056	0.0235	0.0584	<0.0001

As shown in Table 7, the model based on CatBoost-selected features demonstrated a significant improvement over the LightGBM-based model, with an NRI of 1.1004 (95% CI: 0.9785–1.2223; P < 0.0001) and an IDI of 0.041 (95% CI: 0.0235–0.0584; P < 0.0001). Similarly, the CatBoost-based model showed significantly better reclassification than the GBDT-based model (NRI = 0.9938, 95% CI: 0.8661–1.1214; P < 0.0001), although the corresponding IDI did not reach statistical significance (IDI = 0.0057, 95% CI: −0.0151–0.0265; P = 0.591) (Table 8).

**TABLE 8:** NRI and IDI metrics for the CatBoost TEST Factors model and GBDT TEST Factors model.

Row name	Value	SE	Z_value	CI_95_Lower	CI_95_upper	P value
NRI	0.9938	0.0651	15.2613	0.8661	1.1214	<0.0001
IDI	0.0057	0.0106	0.537	−0.0151	0.0265	0.59126

In contrast, the comparison between LightGBM- and GBDT-based models yielded mixed results (Table 9). While the LightGBM model showed a positive NRI (0.2175, 95% CI: 0.074–0.361; P = 0.003), the IDI was significantly negative (−0.0353, 95% CI: −0.0594 to −0.0112; P = 0.004), suggesting that the GBDT-based model provided marginally better discrimination in terms of predicted probability separation.

**TABLE 9:** NRI and IDI metrics for the LightGBM TEST Factors model and GBDT TEST Factors model.

Row name	Value	SE	Z_value	CI_95_lower	CI_95_upper	P value
NRI	0.2175	0.0732	2.9703	0.074	0.361	0.00298
IDI	−0.0353	0.0123	−2.8727	−0.0594	−0.0112	0.00407

In summary, although all logistic models performed well overall, the CatBoost-based model consistently provided superior reclassification compared with the LightGBM- and GBDT-based models. The comparison between LightGBM and GBDT presented a more nuanced picture, indicating a potential trade-off between reclassification and integrated discrimination.

## Discussion

This study developed and validated multiple machine learning models for predicting sarcopenia risk in a community-dwelling elderly population using comprehensive retrospective clinical data from a single center. Our primary findings confirmed that advanced tree-based ensemble algorithms—specifically CatBoost, LightGBM, and GBDT—achieved exceptional discriminative performance, with area under the AUROC values exceeding 0.995. These models significantly outperformed traditional logistic regression across key metrics, including sensitivity and the F1-score. Through SHAP analysis, we identified and ranked pivotal predictive features, thereby enhancing model interpretability and delineating a coherent clinical risk profile for sarcopenia.

The most robust finding was the consistent identification of the SARC-CalF score as the strongest predictor across all top-performing models. This provided powerful, data-driven validation for this simple, low-cost screening tool in community settings (Kera et al., 2022; Li et al., 2025; Tseng et al., 2025). The SARC-CalF score, which combines calf circumference—a surrogate for muscle mass—with the SARC-F questionnaire, was particularly suited for rapid screening in primary and geriatric care (Kera et al., 2022). Our ML-based analysis reinforced its central role and underscored its practical utility for early case identification, especially in primary care.

Beyond the SARC-CalF score, SHAP analysis delineated a risk profile consistent with the core pathophysiology of sarcopenia. Body composition indicators—including body mass index (BMI), and weight—were highly influential predictors. This aligned with the dual diagnostic pillars of sarcopenia: low muscle strength and low muscle mass (Sayer et al., 2024; Chen et al., 2020). The significant contribution of age further solidified its well-established role as a primary non-modifiable risk factor (Cruz-Jentoft and Sayer, 2019) (Huang et al., 2025).

Notably, our models also highlighted conditions associated with chronic inflammation and comorbidity burden, such as osteoarthritis and the total number of chronic diseases. This supported the concept of “secondary” sarcopenia (Barone et al., 2025), wherein chronic disease states and their accompanying inflammatory milieus accelerated muscle protein catabolism and functional decline (Rao et al., 2022; Jang, 2019; Geltser et al., 2019; Jo et al., 2022; Tack et al., 2024; Hosoi et al., 2024; Barbero-Becerra et al., 2020; Wang et al., 2020). Furthermore, sleep duration emerged as a relevant modifiable factor, consistent with growing evidence linking poor sleep quality to impaired muscle metabolism and reduced strength (Matre et al., 2022; Buchmann et al., 2016; Hayashi et al., 2023), and suggesting a potential target for lifestyle intervention (Tung et al., 2023).

A key methodological insight was the clear superiority of complex tree-based ensemble models over traditional logistic regression for this task. Although a logistic regression model built with ML-selected features demonstrated good discrimination (AUC >0.986), it failed completely in sensitivity (recall = 0), rendering it clinically impractical for screening. This performance gap likely stemmed from the ability of algorithms like CatBoost and LightGBM to capture intricate, non-linear interactions among predictors—complexities that the linear assumptions of logistic regression cannot adequately model (Nick and Campbell, 2007). To bridge predictive accuracy and clinical interpretability, we adopted a hybrid strategy, leveraging ML for feature selection and then incorporating the top predictors into a logistic regression framework to construct visually interpretable nomograms. This yielded a practical, points-based scoring system that retains a strong connection to the predictive patterns discovered by the ML algorithms.

The robustness and clinical utility of our models were reinforced by rigorous validation. Five-fold cross-validation during development ensured stable performance and mitigated overfitting. Decision curve analysis (DCA) demonstrated that using any of the top 3 ML models for risk stratification provided greater net clinical benefit across a wide range of decision thresholds than simplistic “treat-all” or “treat-none” strategies. This quantified their value in supporting personalized clinical decision-making. Furthermore, excellent calibration, in which predicted probabilities closely matched observed outcomes, confirmed the reliability of the models’ risk estimates for clinical application.

Several limitations must be acknowledged. First, the single-center, retrospective design may limit generalizability. External validation in independent, multi-center, prospective cohorts from diverse settings was essential. Second, while class imbalance was addressed using SMOTE, the artificially balanced prevalence in the training set necessitated cautious interpretation and underscored the need for validation in populations with natural prevalence rates. Third, our analysis was constrained to routinely collected clinical and demographic variables. Future research could enhance predictive power and biological insight by integrating additional biomarkers (e.g., inflammatory cytokines, hormonal profiles), genetic data, detailed dietary records, and objective physical activity measures. The mechanisms linking inflammation, comorbidities, lifestyle, and sarcopenia warrant further investigation through longitudinal and experimental studies.

## Conclusion

In conclusion, this study demonstrates that machine learning models, particularly tree-based ensembles like CatBoost and LightGBM, can achieve high-performance, well-calibrated prediction of sarcopenia risk in community-dwelling older adults. The analysis reaffirms the paramount importance of the SARC-CalF score alongside core factors like body composition, age, and comorbidity burden. The developed nomograms offer a practical, interpretable tool for individualized risk assessment. By enabling earlier identification of high-risk individuals, this ML-augmented approach can facilitate timely interventions, optimize resource allocation in community geriatric care, and improve patient outcomes. Future work should prioritize external validation, integration of multimodal data, and implementation into user-friendly clinical decision-support systems.

## Data Availability

The original contributions presented in the study are included in the article/supplementary material, further inquiries can be directed to the corresponding authors.
